# Respiratory Pathogen Coinfections in SARS-CoV-2-Positive Patients in Southeastern Wisconsin: A Retrospective Analysis

**DOI:** 10.1128/Spectrum.00831-21

**Published:** 2021-10-20

**Authors:** Samantha J. Scott, Beth Pfotenhauer, Joshua J. Weiner, Jordan Hilleshiem, Manjeet Khubbar, Sanjib Bhattacharyya

**Affiliations:** a City of Milwaukee Health Department Laboratory, Milwaukee, Wisconsin, USA; Houston Methodist Hospital

**Keywords:** COVID-19, coinfections, polymicrobial infections, public health, respiratory pathogen panel, SARS-CoV-2

## Abstract

Severe acute respiratory syndrome coronavirus 2 (SARS-CoV-2), the etiological agent of coronavirus disease 2019 (COVID-19), has infected all age groups and disproportionately impacted vulnerable populations globally. Polymicrobial infections may play an important role in the development of SARS-CoV-2 infection in susceptible hosts. These coinfections may increase the risk of disease severity and pose challenges to the diagnosis, treatment, and prognosis of COVID-19. There have been limited SARS-CoV-2 coinfection studies. In this retrospective study, residual nucleic acid extracts from 796 laboratory-confirmed COVID-19-positive specimens, collected between March 2020 and February 2021, were analyzed using a Luminex NxTAG respiratory pathogen panel (RPP). Of these, 745 returned valid results and were used for analysis; 53 (7.1%) were positive for one or more additional pathogens. Six different respiratory viruses were detected among the 53 SARS-CoV-2-positive patient specimens, and 7 of those specimens tested positive for more than one additional respiratory virus. The most common pathogens include rhinovirus/enterovirus (RV/EV) (*n* = 22, 41.51%), human metapneumovirus (hMPV) (*n* = 18, 33.9%), and adenovirus (*n* = 12, 22.6%). Interestingly, there were no SARS-CoV-2 coinfections involving influenza A or influenza B in the study specimens. The median age of the SARS-CoV-2-positive patients with coinfections was 38 years; 53% identified as female, and 47% identified as male. Based on our retrospective analysis, respiratory coinfections associated with SARS-CoV-2-positive patients were more common in young children (≤9 years old), with white being the most common race. Our findings will likely prompt additional investigation of polymicrobial infection associated with SARS-CoV-2 during seasonal respiratory pathogen surveillance by public health laboratories.

**IMPORTANCE** This examination of respiratory pathogen coinfections in SARS-CoV-2 patients will likely shed light on our understanding of polymicrobial infection associated with COVID-19. Our results should prompt public health authorities to improve seasonal respiratory pathogen surveillance practices and address the risk of disease severity.

## INTRODUCTION

In December of 2019, an outbreak of a novel coronavirus, now called severe acute respiratory syndrome coronavirus 2 (SARS-CoV-2), was detected in Wuhan, China (1). SARS-CoV-2 belongs to the genus β-coronavirus, which includes human coronavirus OC43, human coronavirus HKU1, severe acute respiratory syndrome coronavirus (SARS-CoV), and Middle East respiratory syndrome coronavirus (MERS-CoV) (2, 3). It is similar to other SARS-like β-coronaviruses in bats, but it is distinct from SARS-CoV and MERS-CoV (2). The first case of SARS-CoV-2 in the United States was identified in Washington State in January 2020 (4). Not long after, the first documented case of person-to-person transmission of SARS-CoV-2 in the United States occurred, in late January 2020 (5). SARS-CoV-2 has spread globally, including to every state in the United States, and the illness it causes (coronavirus disease 2019 [COVID-19]) was given pandemic status by the World Health Organization on 11 March 2020 (6). As of 25 June 2021, the COVID-19 pandemic is ongoing, and SARS-CoV-2 has caused 612,507 cases of COVID-19 and 7,280 deaths in the state of Wisconsin (7). Prior to COVID-19 gaining pandemic status, reports out of Wuhan, China, described very low numbers of coinfections with other respiratory pathogens during SARS-CoV-2 infection (8, 9). Currently, there is limited information on the integration of SARS-CoV-2 into existing circulating infection patterns.

The clinical relevance of respiratory pathogen coinfections in association with disease progression remains unclear. The individual respiratory pathogens in a coinfected patient should be considered independently in determining the pathological and clinical significance (10). Due to limited testing resources in the early pandemic, and because coinfections were thought to be uncommon, many clinical laboratories only tested for other respiratory pathogens on SARS-CoV-2-negative specimens or elected not to test for other respiratory pathogens on SARS-CoV-2-positive specimens (11, 12). During the initial phase of the pandemic, the testing algorithms in the United States were based on the Centers for Disease Control and Prevention (CDC) recommendations at the time (12).

In an effort to gain further insight, the Milwaukee Health Department Laboratory (MHDL) performed a retrospective analysis of SARS-CoV-2-positive specimens from southeastern Wisconsin to determine the prevalence of coinfections with other respiratory pathogens.

## RESULTS

### Study specimen characteristics.

MHDL evaluated 796 residual nucleic acid extracts from upper respiratory specimens that had collection dates ranging from March 2020 through February 2021. Of the 796 specimens tested, 7.1% (53/745) were positive for one or more additional pathogens, and 692 (92.9%) tested negative. The remaining 51 (6.5%) returned invalid results and were not considered for additional analysis in this study. The invalid results were most often caused by internal control (MS2) failure (*n* = 46) and low bead count (*n* = 4). One specimen was invalidated due to abnormal signals.

The majority of the specimens tested were collected between March 2020 and May 2020 (*n* = 350, 47%) and September 2020 through November 2020 (*n* = 363, 48.7%). These specimens were collected in a variety of settings and categorized based on the type of submitter. The submitter categories include specimens collected under the direction of the Milwaukee Health Department (MHD) and other local health departments (LHDs) (*n* = 286, 38.4%); hospitals and reference laboratories (*n* = 195, 26.2%); local university student populations (*n* = 99, 13.3%); correctional facilities or other congregate living facilities (CLFs), including staff (*n* = 96, 12.9%); long-term care facilities (LTCFs), including staff (*n* = 41, 5.5%); a county medical examiner’s office (*n* = 24, 3.2%); and local free clinics (*n* = 4, 0.5%).

### Respiratory pathogen and SARS-CoV-2 coinfection patient demographics.

**FIG 1:**
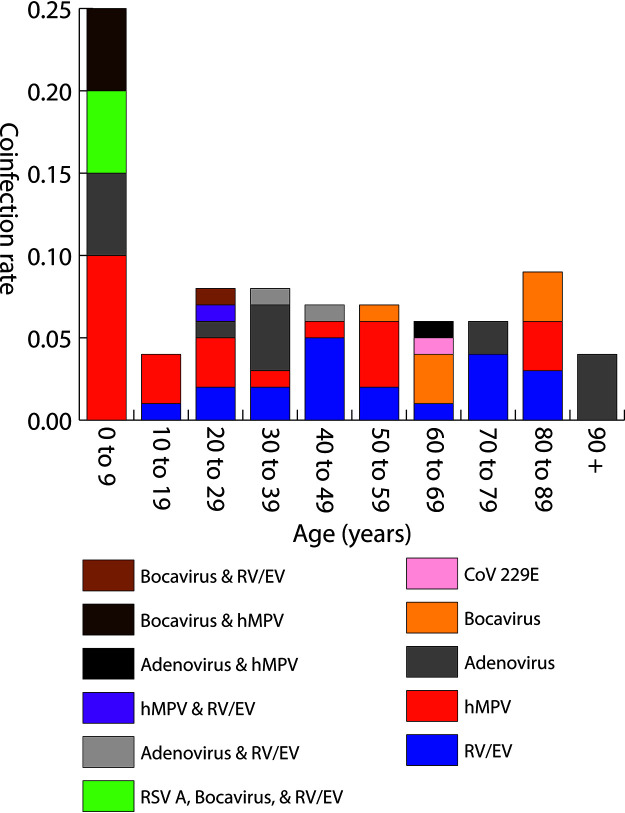
Rate of SARS-CoV-2 and respiratory pathogen coinfections detected within age groups, including pathogen(s) detected. RV/EV, rhinovirus/enterovirus; hMPV, human metapneumovirus; CoV 229E, human coronavirus 229E; RSV A, respiratory syncytial virus A. Total number of valid specimens per group: ≤9 (*n* = 21), 10 to 19 (*n* = 73), 20 to 29 (*n* = 153), 30 to 39 (*n* = 111), 40 to 49 (*n* = 95), 50 to 59 (*n* = 97), 60 to 69 (*n* = 79), 70 to 79 (*n* = 55), 80 to 89 (*n* = 37), ≥90 (*n* = 24).

The patient demographics are listed in Table 1. Patients with coinfections did not differ considerably in gender identity from those positive for SARS-CoV-2 alone. Of the 53 patients who were positive for respiratory viral coinfection, 52.8% (*n* = 28) identified as female, while 47.2% (*n* = 25) identified as male. The median age of the SARS-CoV-2-positive patients coinfected with other respiratory pathogens was 38 years. Significant differences were noted for viral coinfection with SARS-CoV-2 in age distribution (*P < *0.0005), and children under 10 years old were likely to have a higher rate of coinfection than other age groups. Of the SARS-CoV-2-positive specimens in this age group, 23.8% were also positive for one or more respiratory viruses. One specimen collected from the ≤9-year age group was positive for 3 respiratory viruses (respiratory syncytial virus A, rhinovirus/enterovirus, and human bocavirus) in addition to SARS-CoV-2 (Fig. 1).

**TABLE 1:** Demographics of patients with SARS-CoV-2 coinfections^*a*^

Characteristic(s)	Data for:^*b*^		
	SARS-CoV-2 only	SARS-CoV-2 + coinfection	Total
Gender			
Female	343 (49.6)	28 (52.8)	371 (49.8)
Male	333 (48.1)	25 (47.2)	358 (48.1)
Not defined	16 (2.3)	0	16 (2.1)
Median age (yr [range])	41.5 (7 mo to 99)	38 (3 to 102)	41 (7 mo to 102)
Age groups (yr)			
≤9	16 (2.3)	5 (9.4)	21 (2.8)
10–19	70 (10.1)	3 (5.7)	73 (9.8)
20–29	142 (20.5)	11 (20.8)	153 (20.5)
30–39	103 (14.9)	8 (15.1)	111 (14.9)
40–49	88 (12.7)	7 (13.2)	95 (12.8)
50–59	90 (13)	7 (13.2)	97 (13)
60–69	74 (10.7)	5 (9.4)	79 (10.6)
70–79	52 (7.5)	3 (5.7)	55 (7.4)
80–89	34 (4.9)	3 (5.7)	37 (5)
≥90	23 (3.3)	1 (1.9)	24 (3.2)
Race			
White	304 (43.9)	33 (62.3)	337 (45.2)
Black/African American	134 (19.4)	7 (13.2)	141 (18.9)
Asian	5 (0.7)	0	5 (0.7)
Native American	1 (0.1)	1 (1.9)	2 (0.3)
Native Hawaiian	1 (0.1)	0	1 (0.1)
Mixed race	1 (0.1)	0	1 (0.1)
Not provided/other	246 (35.6)	12 (22.6)	258 (34.6)
Ethnicity			
Not Hispanic or Latino	302 (43.6)	31 (58.5)	333 (44.7)
Hispanic or Latino	58 (8.4)	5 (9.4)	63 (8.5)
Not provided	332 (48)	17 (32.1)	349 (46.8)
Specimen submitter types			
Local health department	260 (37.5)	26 (49.1)	286 (38.4)
Hospital/reference laboratory	184 (26.6)	11 (20.7)	195 (26.2)
University	98 (14.2)	1 (1.9)	99 (13.3)
Corrections or other CLF	92 (13.3)	4 (7.5)	96 (12.9)
Long-term care facility	40 (5.8)	1 (1.9)	41 (5.5)
Medical examiner	14 (2)	10 (18.9)	24 (3.2)
Free clinic	4 (0.6)	0	4 (0.5)

a SARS-CoV-2 only, *N *= 692; SARS-CoV-2 + coinfection, *N *= 53; total, *N *= 745.

b Except where otherwise indicated, all data are presented as *n* (%).

Based on the racial distribution, 62.3% (*n* = 33) of coinfection-positive patients identified as white (*P* < 0.05), 13.2% (*n* = 7) identified as Black or African American, 1.9% (*n* = 1) identified as Native American, and 22.6% (*n* = 12) of patients either did not identify a race or indicated that they identified as another race on their specimen requisition. Patients who tested positive for other respiratory pathogens in addition to SARS-CoV-2 did not differ notably in ethnicity from those infected with SARS-CoV-2 monoinfection. Ethnicity was documented for 67.9% (*n* = 36) of the coinfection-positive specimens, of which 58.5% (*n* = 31) identified as “not Hispanic or Latino,” 9.4% (*n* = 5) identified as Hispanic or Latino, and 32.1% (*n* = 17) did not provide an ethnicity.

**FIG 2:**
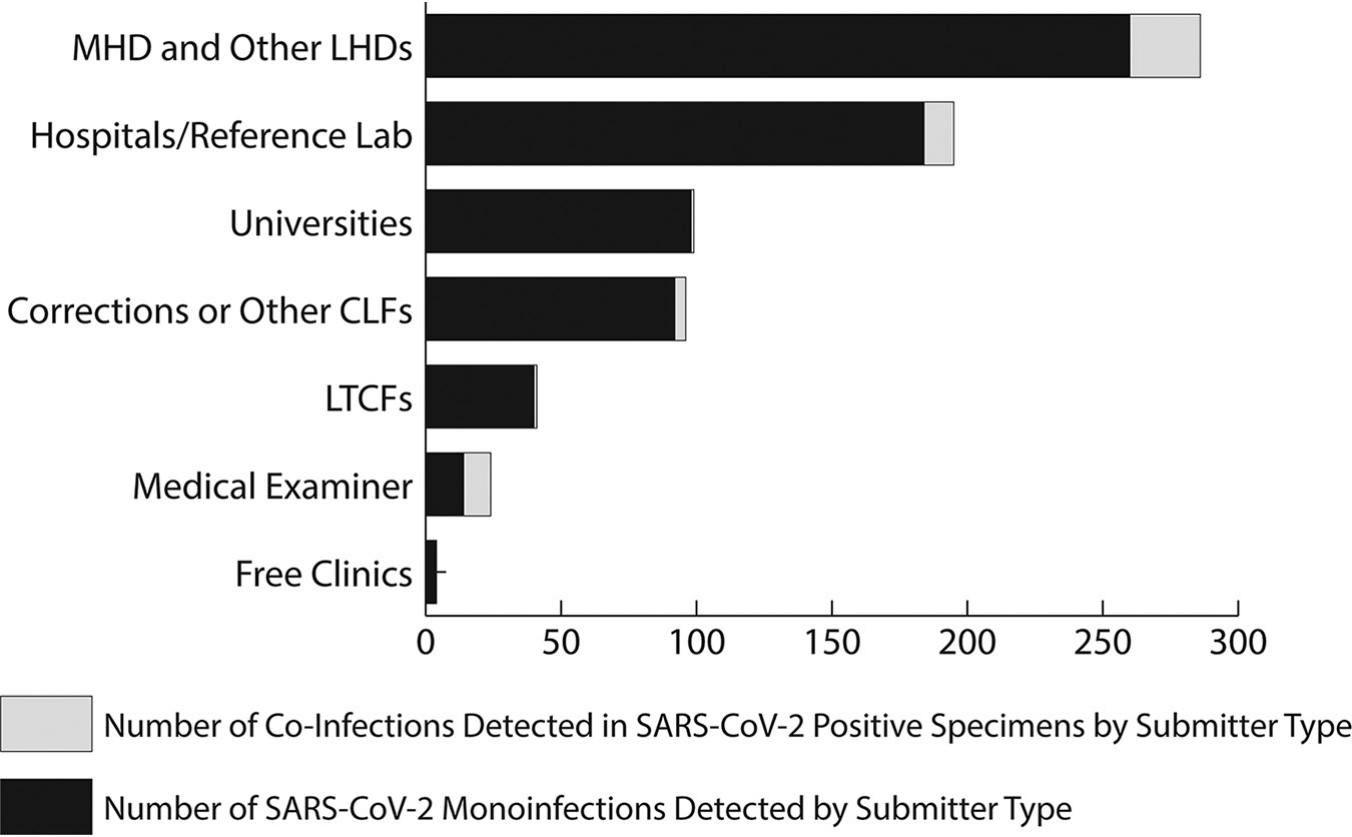
Distribution of positive SARS-CoV-2 samples received by submitter type, including coinfections detected. Submitter categories: Milwaukee Health Department (MHD) and other local health departments (LHDs), hospitals and reference laboratories, local universities, correctional facilities or other congregate living facilities (CLFs), long-term care facilities (LTCFs), medical examiners, and free clinics.

A high positivity rate (*n* = 10/24, 41.7%; *P* < 0.0005) of SARS-CoV-2 coinfections was documented in the respiratory specimens submitted from the medical examiner’s office compared with other facilities (correctional facilities or other congregate living settings [*n* = 4/96, 4.1%] and universities [*n* = 1/99, 1%]). Interestingly, 5 specimens from the medical examiner’s officer tested positive for human metapneumovirus (Fig. 2).

### Distribution of non-SARS-CoV-2 respiratory viruses.

**FIG 3:**
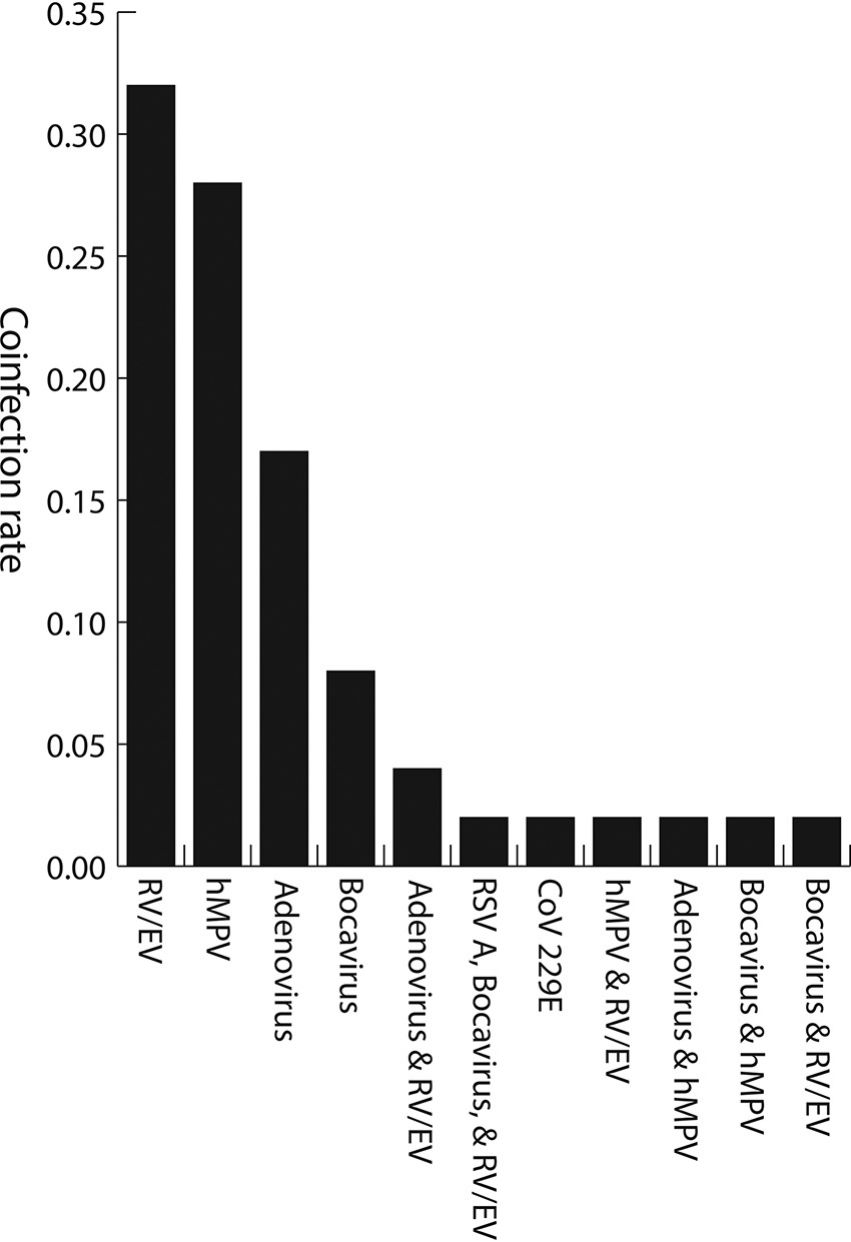
Rate of respiratory viruses detected in SARS-CoV-2-positive specimens. RV/EV, rhinovirus/enterovirus; hMPV, human metapneumovirus; CoV 229E, human coronavirus 229E; RSV A, respiratory syncytial virus A.

Six other respiratory viruses (rhinovirus/enterovirus, human metapneumovirus, adenovirus, human bocavirus, respiratory syncytial virus A, and non-SARS [seasonal] human coronavirus 229E [CoV 229E]) were detected in 53 SARS-CoV-2-positive specimens. Seven of the fifty-three specimens had coinfections with more than one respiratory viral pathogen (Fig. 3). The most common coinfections were rhinovirus/enterovirus (*n* = 22, 41.51%), human metapneumovirus (*n* = 18, 33.9%), and adenovirus (*n* = 12, 22.6%). Interestingly, none of the specimens tested positive for influenza A or influenza B with SARS-CoV-2.

## DISCUSSION

MHDL has been receiving specimens for SARS-CoV-2 diagnosis from a diverse population in southeastern Wisconsin throughout this pandemic. Based on this retrospective study (March 2020 to February 2021), 7.1% of SARS-CoV-2-positive specimens tested positive for one or more respiratory viral pathogens. Higher rates of coinfection were documented on specimens submitted by the medical examiner, in children ≤9 years old, and in those who identified their race as white. None of the SAR-CoV-2-positive specimens tested positive for influenza A/B.

Based on the specimen submitter distribution, our study population includes MHD, other local health department jurisdictions, a county medical examiner, academic universities, hospitals and reference laboratories, long-term care facilities, correctional facilities, and other congregate living settings. Data from this retrospective study reinforce the current CDC SARS-CoV-2 testing guidance, which documents that persons experiencing symptoms need to test and/or self-isolate for 10 days after symptom onset, and persons exposed to a known COVID-19 case need to be tested for SARS-CoV-2 (13). If health care providers were to follow outdated testing algorithms that exclude SARS-CoV-2 testing based on positive results for other respiratory pathogens, the clinical outcome could be devastating (9, 12, 14).

Several articles have reported the possible concomitant presence of respiratory pathogens in subjects with COVID-19. Viral and bacterial pathogens have been described in case reports, case series, and cohorts from low (1.4 to 2.99%) to higher prevalence rates (20.7 to 52.8%), respectively (11, 15–17). In our study, a coinfection rate of 7.1% was found.

Our study differs from other SARS-CoV-2 coinfection studies with reference to the sample size, demographic information, and analytes tested (14, 18–21). Unlike in other published studies, none of our study specimens were screened for a wide array of respiratory bacterial and fungal pathogens (18, 22). Therefore, the percentage prevalence of other respiratory pathogens in the SARS-CoV-2-positive specimens was likely underestimated.

In our study, rhinovirus/enterovirus (RV/EV), human metapneumovirus (hMPV), and adenovirus were the most frequently detected respiratory virus coinfections in the SARS-CoV-2-positive specimens. The rates of SARS-CoV-2 and respiratory virus coinfection in our study population are concordant with other published literature (11, 14–17, 19, 22, 23). None of our specimens tested positive for influenza A or B, and one patient was likely to be coinfected with non-SARS (seasonal) human coronavirus 229E (CoV 229E). The absence of influenza virus coinfections with SARS-CoV-2 may be attributable to the overall decrease in influenza activity observed globally since the start of the COVID-19 pandemic, and it is conceivable that the mitigation and control measures put into place to check the COVID-19 pandemic also played a key role in the overall reduction of influenza cases (24–27).

In our study, respiratory coinfections in SARS-CoV-2 were more common in young children (≤9 years old). As reported by current published literature, children (<18 years old) can be coinfected with SARS-CoV-2 and other respiratory pathogens such as human metapneumovirus, adenovirus, respiratory syncytial virus, Mycoplasma pneumoniae, and influenza A and B (22, 23, 28–30).

Based on the racial distribution of those who tested positive for SARS-CoV-2 and other respiratory pathogens, 62.3% were white. The data may be underestimated, as the majority of our study population declined to identify their race or the race selection question was not listed on the specimen requisition form. Further studies are warranted exploring the correlation of racial distribution with SARS-CoV2-associated coinfections. The U.S. Veteran’s Health Administration documented that Black or African American individuals were more likely to have SARS-CoV-2 coinfections compared with individuals infected only with non-SARS-CoV-2 pathogens (11).

The SARS-CoV-2-positive specimens submitted by a county medical examiner’s office had a high rate (41.7%) of respiratory viral coinfection. Studies documenting the postmortem detection of coinfections with SARS-CoV-2 have been limited during the pandemic (31, 32). This in part is due to required enhanced precautions, biosafety measures, and the limited numbers of autopsies being conducted (32–35). In the United States, the CDC strongly recommends postmortem testing for additional respiratory pathogens, but this is not a mandated requirement (36).

Another interesting aspect of our study is that it includes data beyond May 2020. A majority of the published studies documented SARS-CoV-2 coinfections in specimens collected from December 2019 through May 2020 (9, 11, 14–19, 22, 23, 37, 38). Our study data were collected from March 2020 through February 2021.

As a public health laboratory, MHDL has limited access to patient medical charts; therefore, our acquisition of most demographic information relied on the available data provided on the specimen requisition. Other comorbidities in our study population may have compromised the results. The study was limited to a subset of the population from the southeastern region of Wisconsin; therefore, the data may not reflect the true prevalence of the other respiratory pathogen coinfections with SARS-CoV-2 in this region. Large longitudinal surveillance studies are warranted to establish the true prevalence of respiratory pathogen coinfections with SARS-CoV-2 in specific populations over a specific period of time. Subsequently, our preliminary data may be extrapolated for southeastern Wisconsin to determine the seasonal incidence of respiratory pathogens in the adult and pediatric populations to unveil the clinicopathological impact of SARS-CoV-2 with other respiratory viruses. Based on a clinical and epidemiological perspective, the ability of the laboratory to screen for other respiratory pathogens during a pandemic could play an important role in appropriate diagnosis and treatment.

## MATERIALS AND METHODS

### Specimen collection.

Residual nucleic acid extracts from previously tested SARS-CoV-2 specimens were used in this study. The original respiratory specimens (nasal swab, nasopharyngeal swab, combined nasopharyngeal/oropharyngeal swabs, oropharyngeal swab, and sputum) were collected for SARS-CoV-2 diagnosis between March 2020 and February 2021 from a diverse population in southeastern Wisconsin. The specimens were collected according to CDC guidelines and transported to MHDL in a timely manner (39). All patient demographic information was provided by submitters via the MHDL Microbiology Requisition form. These data were entered into MHDL’s laboratory information system at the initial time of the SARS-CoV-2 diagnostic testing and were later isolated, deidentified, and organized in spreadsheet format for populating other respiratory coinfection data sets. Upon completion of SARS-CoV-2 testing, aliquots of the SARS-CoV-2-positive original specimen (respiratory swabs in viral transport medium or saline, or sputum), as well as residual nucleic acid extracts, were immediately frozen in our laboratory specimen repository at −70°C according to standard practice to ensure integrity (40, 41). The archived nucleic acids were later withdrawn from MHDL’s specimen repository for further analysis.

### Ethics.

All residual, deidentified nucleic acid specimens used in this study were collected for public health surveillance purposes only and were exempt from human subject research as per the Milwaukee Health Department Laboratory biosafety and ethics committee guidelines.

### Nucleic acid extraction and respiratory pathogen panel analysis.

Nucleic acid extractions had been previously performed using automated extraction instruments, including the Maxwell rapid sample concentrator (RSC) platform (Promega, Madison, WI), NucliSens easyMAG and EMAG platforms (bioMérieux, Boxtel, The Netherlands), and EZ1 Advanced XL (Qiagen, Valencia, CA). These extraction platforms were approved for use in both the CDC 2019-nCoV and the CDC FLU SC2 multiplex assays authorized by the Food and Drug Administration (FDA) Emergency Use Authorization (EUA) (40, 42). These assays require nucleic acid extraction controls for every batch of specimens in order to report valid clinical results. For research purposes, MHDL has determined that spiking previously extracted bacteriophage MS2 RNA into the residual nucleic acids as a reverse-transcription and amplification control performs adequately.

MS2 control nucleic acids were extracted using the NucliSens easyMAG (bioMérieux) specified in the NxTAG respiratory pathogen panel (RPP) (Luminex Molecular Diagnostics, Austin, TX) package insert and stored in the same manner as the clinical specimen nucleic acids until use (43). Residual nucleic acids from 796 specimens that had previously tested positive for SARS-CoV-2 viral RNA were spiked with a 1:10 dilution of previously extracted MS2 nucleic acid and used for multiplex pathogen analysis using the NxTAG RPP assay. This *in vitro* diagnostic (IVD)-cleared assay detects 20 upper and lower respiratory pathogens and incorporates multiplex reverse transcriptase PCR (RT-PCR) with a proprietary universal tag sorting system to detect respiratory pathogen targets. The results were then analyzed using the NxTAG respiratory pathogen panel assay file for SYNCT software according to the manufacturer’s recommendations (43).

### Statistical analysis.

Coinfection was determined by the detection of SARS-CoV-2 with one or more additional respiratory pathogens in the same specimen. The SARS-CoV-2 and respiratory pathogen coinfection data were then analyzed for the frequency and patterns of SARS-CoV-2 detection with other respiratory pathogens. Categorical variables were expressed as numbers and percentages, while continuous variables were represented as median and range. Fisher’s exact test was performed on the age group, gender, race, ethnicity, and submitter category versus coinfection contingency table manually using command line R language with 2000 Monte Carlo calculations to simulate *P* values (R Foundation for Statistical Computing, Vienna, Austria). The results were considered statistically significant for two-sided *P* values of <0.05.
